# Clinical characteristics of choroidal microvasculature dropout in normal-tension glaucoma versus nonarteritic anterior ischemic optic neuropathy: an optical coherence tomography angiography study

**DOI:** 10.1038/s41598-021-00868-5

**Published:** 2021-11-01

**Authors:** Joong Won Shin, Jin Yeong Lee, Byung Joo Lee, Hyun Taek Lim, Michael S. Kook

**Affiliations:** Department of Ophthalmology, Asan Medical Center, College of Medicine, University of Ulsan, 388-1 Pungnap-2-dong, Songpa-gu, Seoul, 138-736 Korea

**Keywords:** Ocular hypertension, Glaucoma

## Abstract

The present study investigated the characteristics of choroidal microvasculature dropout (CMvD) in eyes with nonarteritic anterior ischemic optic neuropathy (NAION) versus those in eyes with normal-tension glaucoma (NTG). This study included 27 NAION, 27 NTG, and 27 healthy control subjects. CMvD was observed in 15 eyes (55.6%) of the NAION group and 20 (74.1%) of the NTG group. The area and angular width of CMvD were significantly greater in eyes with NAION (0.278 ± 0.172 mm^2^ and 86.5 ± 42.3°) than in those with NTG (0.138 ± 0.068 mm^2^ and 35.1 ± 16.2°, *p* = 0.002 and *p* < 0.001, respectively). CMvD in eyes with NAION were distributed in 120–250° and most frequently located at the temporal region, while CMvD in eyes with NTG showed double peaks at 220–280° and 110–140° and most frequently located at the inferotemporal region. The factors associated with the discrimination of NAION from NTG were greater area of CMvD (OR, 1.181; 95% CI, 1.021–1.366; *p* = 0.025) and location closer to the temporal region of the CMvD (OR, 0.904; 95% CI, 0.838–0.975; *p* = 0.009). The clinical characteristics of CMvD differed between eyes with NAION and those with NTG. Optical coherence tomography angiography may provide an additional approach to differentiating glaucoma from NAION.

## Introduction

Nonarteritic anterior ischemic optic neuropathy (NAION) is the most common type of non-glaucomatous optic neuropathy in middle-aged and elderly patients and the second most common optic neuropathy after glaucoma^1^. Although the exact pathogenetic mechanism of NAION remains unclear, it is presumed to result from transient nonperfusion or hypoperfusion within the optic nerve head (ONH)^2^. The main source of blood supply to the ONH is the short posterior ciliary artery (SPCA)^3^, whose circulation is reduced in patients with NAION^4^. Glaucomatous optic neuropathy (GON) is associated with the vascular insufficiency of ONH, represented by peripapillary choroidal microvasculature dropout (CMvD) according to optical coherence tomography angiography (OCTA)^5^. Based on an indocyanine green dye study, CMvD corresponds to a true filling defect of the peripapillary choroidal circulation, which is downstream from the SPCA^6^. Although NAION and GON may share similar origins of vascular impairment, the presence of CMvD in eyes with NAION remains unexplored.

The clinical manifestation of NAION in the acute phase, such as painless loss of vision occurring over hours to days or optic disc oedema, could help distinguish it from GON. In the chronic phase, however, differentiating NAION from GON is more challenging because the pattern of retinal nerve fiber layer (RNFL) loss and visual field (VF) defects can appear similar between the two diseases^7–9^. Previous studies reported that the differences in RNFL thickness and peripapillary vessel density (pVD) between eyes with NAION and those with GON were variable and inconclusive^7,10,11^. Therefore, there is a need to identify a viable clinical criterion for discriminating NAION from GON. We hypothesised that CMvD would be observed in eyes with NAION since it may be associated with a perfusion insufficiency in ONH. However, the clinical features of CMvD in eyes with NAION may differ from those in eyes with GON. While the primary event in NAION may be a perfusion impairment, the pathogenesis of GON is explained by two principal theories, mechanical and ischemic, while the impact of ischemia may be more prominent in normal-tension glaucoma (NTG) than in primary open-angle glaucoma (POAG)^12,13^. Therefore, we investigated the presence of CMvD in eyes with NAION and compared their clinical characteristics to those of eyes with NTG.

## Materials and methods

This retrospective cross-sectional study recruited patients with NAION or NTG and healthy controls who visited Asan Medical Center (Seoul, Korea) between January 2019 and December 2020. The Institutional Review Board of Asan Medical Center approved this study, and all procedures were performed in accordance with the principles of the Declaration of Helsinki. The waiver of informed consent was granted by the Institutional Review Board of Asan Medical Center due to the retrospective study design.

All participants underwent complete ophthalmologic examinations as follows:^14^ the measurement of best-corrected visual acuity, intraocular pressure (IOP) using Goldmann applanation tonometry, refractive error using an autorefractor (KR-890; Topcon Corp, Tokyo, Japan), slit-lamp biomicroscopy, gonioscopy, dilated colour fundus photography, stereoscopic optic disc photography, red-free fundus photography, standard automated perimetry (Humphrey Field Analyzer with Swedish Interactive Threshold Algorithm standard 24-2 test; Carl Zeiss Meditec, Dublin, CA, USA), ONH and RNFL imaging on spectral-domain optical coherence tomography (SD-OCT; Cirrus HD-OCT, Carl Zeiss Meditec), and peripapillary angiography with OCTA (AngioVue; Optovue Inc, Fremont, CA, USA). The inclusion criteria were as follows: a spherical equivalent between − 8.0 and + 3.0 diopters (D) and a cylinder correction within + 3.0 D, a normal anterior chamber and open-angle on slit-lamp and gonioscopic examinations, and two reliable VF test results with a false-positive error < 15%, false-negative error < 15%, and fixation loss < 20%, and visible the β-zone parapapillary atrophy (β-PPA) on fundus photography.

NAION was defined as the presence of sudden painless vision loss, optic disc oedema with or without superficial haemorrhages at onset, a pale optic disc with complete resolution of optic disc oedema at the time of enrolment, and compatible VF defects. The NAION group included only chronic patients diagnosed at least 6 months prior. Those with suspected arteritic anterior ischemic optic neuropathy with a high erythrocyte sedimentation rate or C-reactive protein level were excluded. NTG was defined as the presence of glaucomatous ONH changes (focal or diffuse neural rim loss, disc haemorrhage, or RNFL defects on red-free photography) with compatible glaucomatous VF defects and no history of untreated maximum IOP ≥ 22 mmHg during an outpatient clinic visit. Glaucomatous VF defects were confirmed by the following: a glaucoma hemifield test result outside normal limits; an abnormal pattern deviation probability plot with three contiguous points with < 5% probability, including at least 1 point with a < 1% probability; and pattern standard deviation outside the 95% normal limits as confirmed by two consecutive reliable tests. The healthy control group included eyes with a best-corrected visual acuity of ≥ 20/30, normal optic disc appearance, IOP ≤ 21 mmHg, and no abnormal VF defects.

After the enrolment of NAION eyes (n = 27), eyes with NTG were selected and matched for VF mean deviation (MD; ≤ 1 dB) to the eyes with NAION since VF damage severity is known to be associated with the presence of CMvD^5^. Then, NTG and healthy control eyes were matched for age (± 5 years) to the eyes with NAION. If multiple NTG and healthy subjects met the conditions for VF MD and age matching, one was randomly selected from each diagnostic group. When both eyes of a participant were eligible, one was selected at random. Patients were excluded from the study if they had a history of an intraocular procedure or surgery except for uncomplicated cataract extraction, history of trauma, retinal vaso-occlusive disease, severe myopic retinal or macular changes that could render the ONH/VF evaluation of glaucoma difficult, or other ocular or systemic disease that may alter the VF test results other than glaucoma and NAION.

### Optical coherence tomography angiography

All participants underwent ONH OCTA imaging of a 4.5 × 4.5 mm^2^ region centerd on the optic disc using an AngioVue OCTA system (version 2018.1.0.37; Optovue Inc.). A split-spectrum amplitude-decorrelation angiography algorithm was used to identify perfused vessels by capturing the dynamic motion of moving red blood cells. Details have been described elsewhere^15,16^. Vessel density (VD) was evaluated within the radial peripapillary capillary slab from the internal limiting membrane to the nerve fiber layer after automated removal of the large vessels using the AngioVue software. PVD was the percentage of measured area occupied by small vessels in a region defined as a 1000-µm-wide elliptical annulus extending from the optic disc boundary. Sectoral pVD was measured at the nasal (N), inferonasal (IN), inferotemporal (IT), temporal (T), superotemporal (ST), and superonasal (SN) regions. Only good-quality images with scan quality ≥ 7 and without poor clarity, motion artifacts, localized weak signal due to floater or posterior vitreous detachment, or segmentation errors were included and analyzed.

### Evaluation of β-PPA area and choroidal microvasculature dropout

First, the β-PPA was defined as an atrophic region of the retinal pigment epithelium resulting in visible sclera and choroidal vessels adjacent to the optic disc. Since the presence of CMvD is associated with the size of β-PPA, the β-PPA area was calculated using Image J software (version 1.52; Wayne Rasband, National Institutes of Health, Bethesda, MD) as reported in the previous study^5^. Eyes without clear optic disc or β-PPA margins were excluded from the study.

CMvD was evaluated within the β-zone parapapillary atrophy (β-PPA) based on the choroidal layer of the en face OCTA image. The β-PPA was defined as an atrophic region of the retinal pigment epithelium resulting in visible sclera and choroidal vessels adjacent to the optic disc. CMvD was defined as the complete loss of the choriocapillaris or microvasculature on a horizontal B-scan and the en face choroidal layer OCTA image^5^. CMvD was required to be present in at least four consecutive horizontal B-scans and be ≥ 200 μm in at least one scan. Two independent observers (J.W.S. and J.Y.L.) blinded to the patients’ clinical data determined the presence and boundary of CMvD, then measured the area and angular width of CMvD using previously described approach^17^. The boundary of the β-PPA for the measurement of choroidal VD was also delineated by the same observers. Disagreements regarding the presence of CMvD between the two observers were resolved by a third adjudicator (M.S.K.). The choroidal VD in the β-PPA was measured as reported in previous study using customised MATLAB software (The MathWorks, Inc., Natick, MA, USA) after removal of the large retinal vessels^18^. Briefly, an automated thresholding algorithm generated a binary image from the choroidal layer of the en face ONH OCTA image. The blood vessels were defined by the pixels with values above the threshold level. The VD was the ratio of the total vessel area over the total area of the region of interest^18^. The average values from the measurements of the two masked observers were used in the analysis to minimize interobserver variation.

### Spectral-domain optical coherence tomography

The RNFL thickness was measured at a 6 × 6 mm^2^ region with the optic disc cube scan using the Cirrus HD-OCT (Carl Zeiss Meditec). The peripapillary RNFL thickness (pRNFLT) was measured at 256 points using the Advanced Export program provided by the manufacturer in a circle with a 3.46-mm diameter. Each point from 256 cpRNFLT measurements was allocated to the six sectors (N, IN, IT, T, ST, and SN), and the cpRNFLT of each sector was calculated by averaging the corresponding points of the 256 cpRNFLT measurements extracted from the Cirrus HD-OCT. Poor quality SD-OCT images with motion artifacts, segmentation failure, or signal strength < 7 were excluded.

### Statistical analysis

Interobserver agreement for determining the presence of CMvD was assessed by kappa statistics. The interobserver reproducibility in the measurements of the area and angular width of the CMvD were evaluated by calculating intraclass correlation coefficients (ICCs). The demographics and clinical characteristics were compared between the NAION and other groups (NTG and control groups) using the independent Student’s t-test for normally distributed data; otherwise, the Mann–Whitney U test was used. Normality was tested using the Shapiro–Wilk test. Categorical variables were compared between the groups using the chi-square test. Factors associated with the discrimination of NAION from NTG were assessed by univariable and multivariable logistic regression analyses. Independent variables with values of *p* < 0.1 in the univariable model were entered to the multivariable model. *p* values < 0.05 were considered statistically significant. The statistical analyses were performed using SPSS (ver. 20.0; IBM Corp., Armonk, NY, USA).

## Results

Of the 39 eyes of 39 NAION patients that initially met the inclusion criteria, 9 (23.1%) and 3 (7.7%) eyes were excluded because of the absence of β-PPA and poor quality of OCTA or SD-OCT images. Therefore, this study included 27 eyes of 27 NAION patients that were age- and VF MD–matched to 27 eyes of 27 NTG patients and age-matched to 27 eyes of 27 healthy control subjects in the final analysis. The interobserver agreement in determining the presence of the CMvD was excellent [κ = 0.915; 95% confidence interval (CI), 0.821–0.974; *p* < 0.001]. The ICCs (95% CI) for the interobserver reproducibility of the measurements of the area and angular width of the CMvD were 0.921 (0.864–0.955), and 0.943 (0.886–0.978), respectively.

Table 1 summarizes the demographic and clinical characteristics of the study participants. CMvD was observed in 15 NAION eyes (55.6%), 20 NTG eyes (74.1%), and none of the control eyes. There was no significant difference in the presence of CMvD between the NAION and NTG eyes. In addition, choroidal VD in the β-PPA did not differ significantly between the two groups, although the NAION and NTG eyes showed significantly reduced choroidal VD compared to the control eyes. However, the area and angular width of CMvD were significantly different between the NAION and NTG eyes. The area and angular width of CMvD in the NAION eyes (0.278 ± 0.172 mm^2^ and 86.5 ± 42.3°) were more than twice as large as those in the NTG eyes (0.138 ± 0.068 mm^2^ and 35.1 ± 16.2°, *p* = 0.002 and *p* < 0.001, respectively).

**Table 1:** Comparisons of the demographic and clinical characteristics among the NAION, NTG, and healthy control eyes.

	NAION group	NTG group	Healthy group	*p* value	*p* value
				NAION versus NTG group	NAION versus healthy group
Number of eyes	27	27	27		
Sex (male/female)	16/11	10/17	11/16	0.102	0.174
Age (years)	65.2 ± 7.8	61.7 ± 11.1	61.6 ± 7.1	0.182	0.079
Refractive error (D)	− 0.16 ± 1.73	− 1.13 ± 2.61	− 0.66 ± 1.59	0.117	0.275
Intraocular pressure (mmHg)	15.6 ± 3.2	14.9 ± 4.8	14.5 ± 2.5	0.547	0.171
Hypertension (n, %)	5 (18.5%)	3 (11.1%)		0.444	
Diabetes (n, %)	3 (11.1%)	2 (7.4%)		0.639	
Hyperlipidemia (n, %)	2 (7.4%)	4 (14,8%)		0.386	
Visual field mean deviation (dB)	− 15.13 ± 8.34	− 15.11 ± 7.57	− 0.08 ± 1.47	0.991	< **0.001**
Cup-to-disc ratio	0.57 ± 0.17	0.78 ± 0.07	0.59 ± 0.11	< **0.001**	0.546
Peripapillary RNFL thickness (μm)\	68.6 ± 11.2	67.4 ± 12.1	89.5 ± 8.5	0.700	< **0.001**
Peripapillary vessel density (%)	32.1 ± 7.4	38.1 ± 7.9	59.4 ± 3.5	**0.006**	< **0.001**
Choroidal vessel density of β-PPA (%)	49.9 ± 5.2	50.0 ± 5.2	59.7 ± 3.2	0.949	< **0.001**
β-PPA area (mm^2^)	1.166 ± 0.410	1.215 ± 0.566	1.369 ± 0.414	0.735	0.089
Presence of CMvD (n, %)	15 (55.6)	20 (74.1)	0	0.154	
CMvD area (mm^2^)	0.278 ± 0.172	0.138 ± 0.068		**0.002**	
CMvD angular width (°)	86.5 ± 42.3	35.1 ± 16.2		< **0.001**	

*β-PPA* β-zone peripapillary atrophy, *CMvD* Choroidal microvasculature dropout, *D* Diopter, *NAION* Nonarteritic anterior ischemic optic neuropathy, *NTG* Normal-tension glaucoma, *RNFL* Retinal nerve fiber layer.

Significant *p* values are shown in bold type.

**Figure 1:**
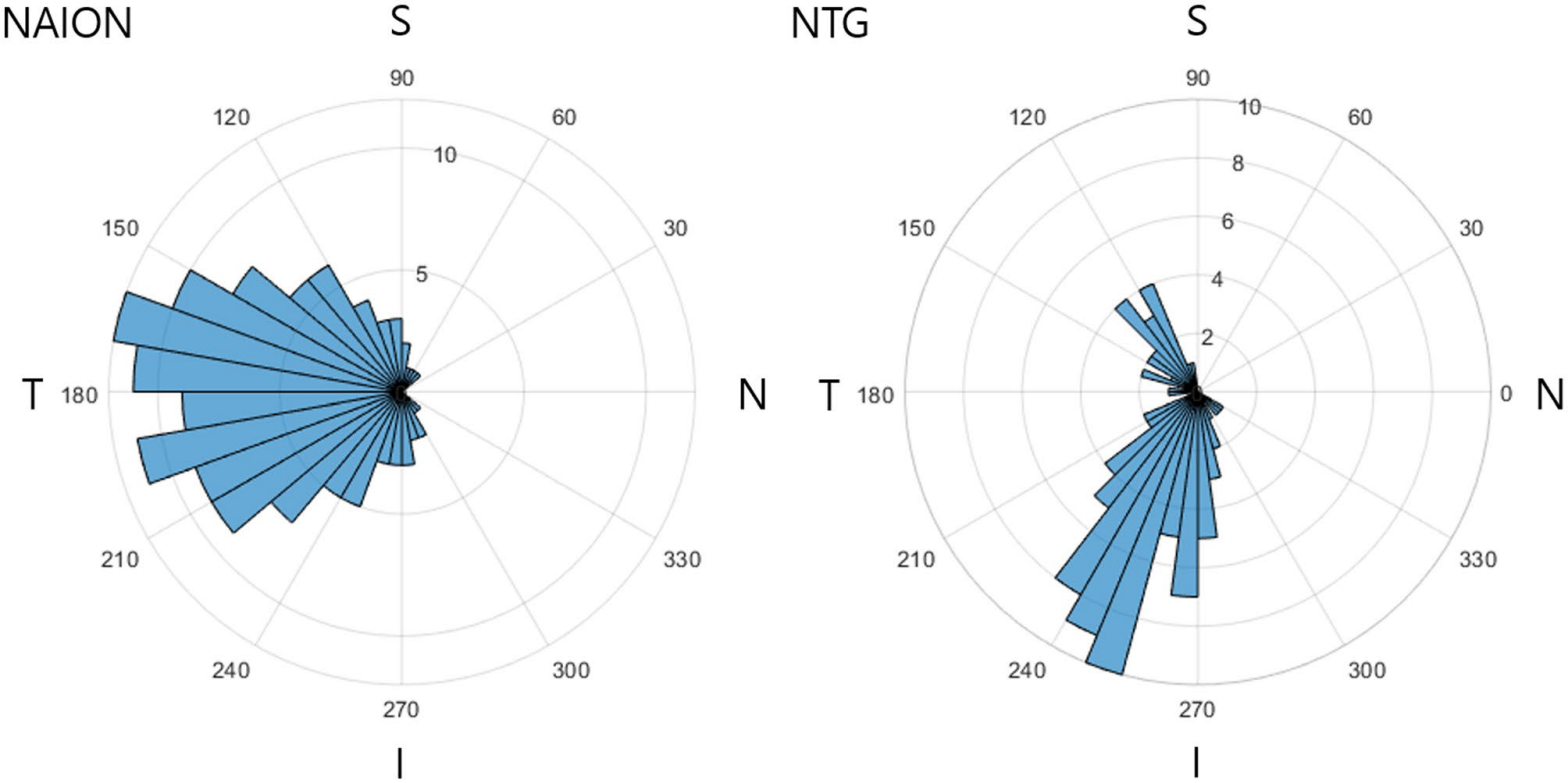
Frequency distribution polar plots of the location of choroidal microvasculature dropout assessed by optical coherence tomography angiography in eyes with nonarteritic anterior ischemic optic neuropathy (NAION) or normal-tension glaucoma (NTG). S, Superior sector; N, Nasal sector; I, Inferior sector; T, Temporal sector.

Figure 1 shows the frequency distribution plots of the CMvD location in eyes with NAION versus those with NTG based on the right eye configuration. The CMvD of eyes with NAION were distributed in a relatively wide range (120–250°) and located most frequently at the temporal region. Contrastingly, the CMvD in eyes with NTG were distributed in a relatively narrow range with double peaks at 220–280° and 110–140° and located most frequently at the inferotemporal region.

**Figure 2:**
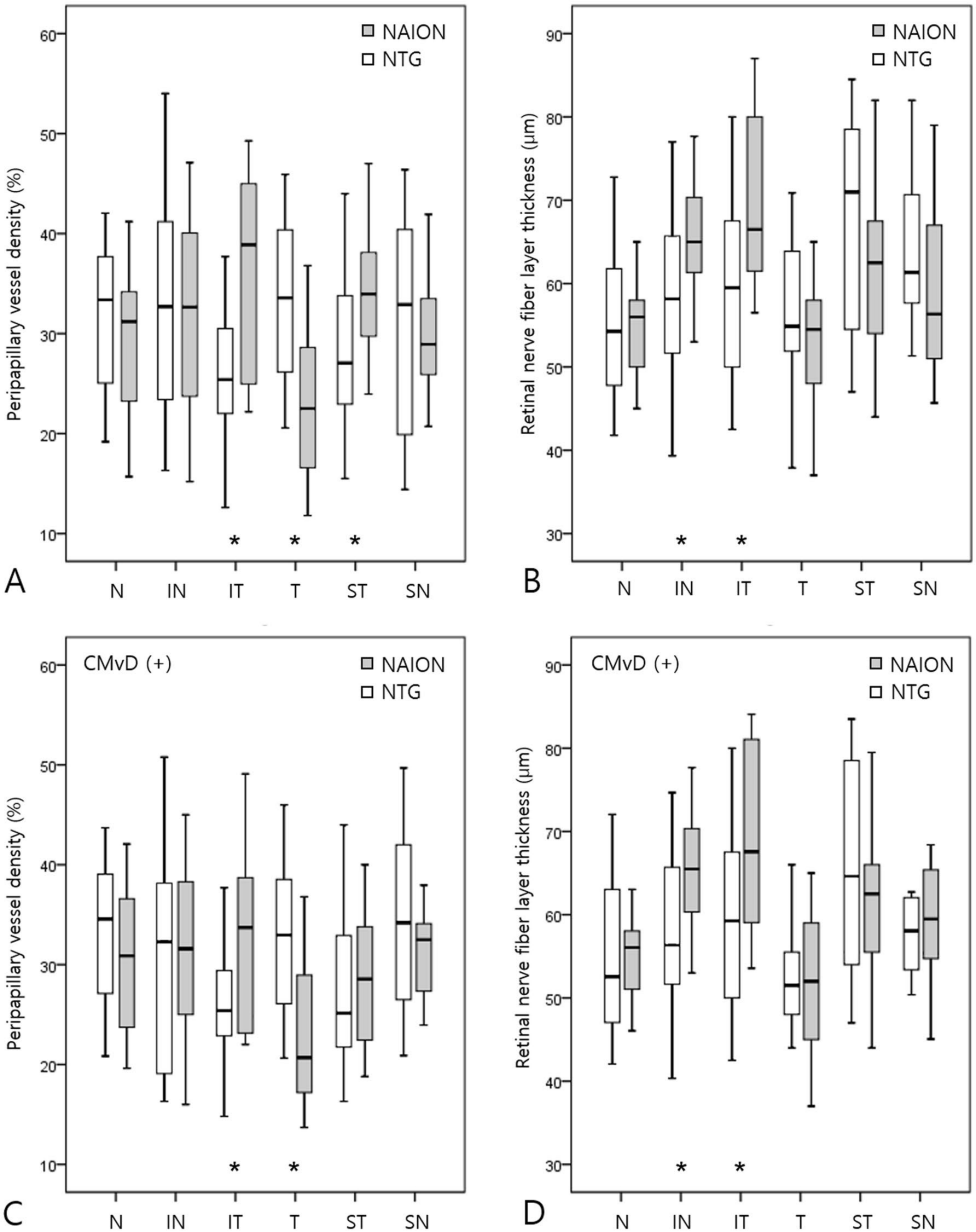
Box plots showing the sectoral peripapillary vessel density (pVD) and peripapillary retinal nerve fiber layer thickness (pRNFLT) of all eyes (n = 54; **A**, **B**) and eyes by subgroup with choroidal microvasculature dropout (CMvD; n = 35; **C**, **D**). Significant differences in pVD and pRNFLT between eyes with nonarteritic anterior ischemic optic neuropathy (NAION) and those with normal-tension glaucoma (NTG) are marked with asterisks.

Global pVD was significantly lower in eyes with NAION than in eyes with NTG (32.1 ± 7.4 vs. 38.1 ± 7.9, *p* = 0.006), but global pRNFLT did not differ significantly between the two groups (Table 1). Figure 2 compares the sectoral pVD and pRNFLT between NAION and NTG eyes. Temporal pVD was significantly lower in eyes with NAION than in those with NTG, whereas inferotemporal and superotemporal pVD were significantly lower in eyes with NTG than in those with NAION. Inferonasal and inferotemporal RNFL thicknesses were significantly lower in eyes with NTG than in those with NAION. (Fig. 2A,B) In a subgroup analysis of eyes with CMvD (n = 35), similar trends were observed in the sectoral pVD and pRNFLT measurements (Fig. 2C,D).

**Figure 3:**
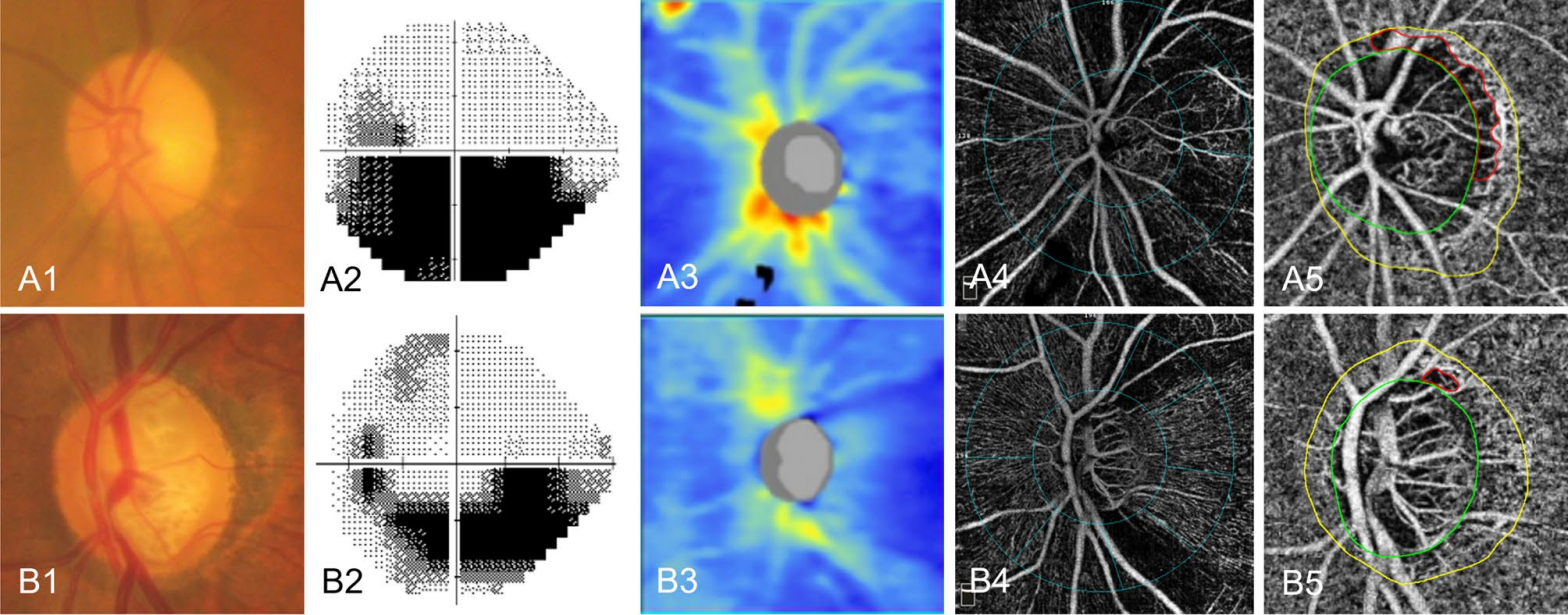
Representative cases of nonarteritic anterior ischemic optic neuropathy (NAION) eye and age- and visual field severity-matched normal-tension glaucoma (NTG) eye showing different clinical characteristics of choroidal microvasculature dropout (CMvD). Color fundus photography of NAION eye (A1) at chronic stage and NTG eye (B1) with corresponding visual field defects (A2, B2); Optical coherence tomography thickness map and superficial vessel density image in the peripapillary area of NAION eye (A3, A4) and NTG eye (B3, B4); En face parapapillary choroidal layer image of optical coherence tomography angiography of NAION eye (A5) and NTG eye (B5), in which the green ellipse and yellow line indicate the optic nerve head and β-zone parapapillary atrophy margin. The CMvD area is enclosed by the red colored border. Eye with NAION (A5) had a larger sized and wider-ranged CMvD involving the temporal region, while eye with NTG (B5) had a smaller sized and narrower-ranged CMvD involving the superotemporal region.

Table 2 shows the factors associated with the discrimination of NAION from NTG. In the univariable logistic regression analysis, older age, lower pVD, greater CMvD area, wider CMvD angular width, and CMvD location closer to the temporal region were significantly associated with the diagnosis of NAION. In the multivariable analysis, the factors associated with the discrimination of NAION from NTG were greater CMvD area [odds ratio (OR), 1.181; 95% CI, 1.021–1.366; *p* = 0.025] and CMvD location closer to the temporal region (OR, 0.904; 95% CI, 0.838–0.975; *p* = 0.009). As illustrative cases, eye with NAION (Fig. 3A) had larger area of CMvD and wider-range CMvD involving the temporal region, while eye with NTG (Fig. 3B) had smaller area of CMvD and narrower-range CMvD involving the superotemporal region despite both eyes having similar age and MD between the two eyes.

**Table 2:** Factors associated with the diagnosis of NAION versus NTG.

	Univariable			Multivariable		
Variables	OR	95% CI	*p* value	OR	95% CI	*p* value
Age (per 1 yr older)	1.096	1.008–1.192	**0.032**	1.057	0.906–1.234	0.479
Refractive error (per 1 D higher)	1.360	0.915–2.021	0.129			
Hypertension, presence	1.818	0.388–8.514	0.448			
Diabetes, presence	1.562	0.240–10.187	0.641			
Hyperlipidemia, presence	0.460	0.077–2.753	0.395			
Intraocular pressure (per 1 mmHg higher)	1.099	0.914–1.321	0.315			
Peripapillary RNFL thickness (per 1 μm thicker)	1.005	0.942–1.072	0.888			
Peripapillary vessel density (per 1% higher)	0.890	0.802–0.989	**0.030**	0.940	0.772–1.144	0.534
Choroidal vessel density of β-PPA (per 1% higher)	1.019	0.888–1.170	0.787			
β-PPA area (per 0.01 mm^2^ larger)	0.998	0.987–1.009	0.729			
CMvD area (per 0.01 mm^2^ larger)	1.123	1.023–1.232	**0.014**	1.181	1.021–1.366	**0.025**
CMvD angular width (per 1° wider)	1.059	1.020–1.100	**0.003**	1.013	0.958–1.070	0.660
CMvD angular location (per 1° away from temporal area)	0.938	0.901–0.976	**0.002**	0.904	0.838–0.975	**0.009**

*β-PPA* β-zone peripapillary atrophy, *CI* Confidence interval, *MvD* Choroidal microvasculature dropout, *NAION* Nonarteritic anterior ischemic optic neuropathy, *NTG* Normal-tension glaucoma, *OR* Odds ratio, *RNFL* Retinal nerve fiber layer.

Boldface *p* values indicate statistical significance.

## Discussion

In this study, CMvD was detected in 55.6% of NAION eyes, which had different CMvD characteristics than NTG eyes. CMvD was first identified and described in eyes with GON, with which several studies have emphasised its clinical relevance^17,19–23^. As studies exploring the presence of CMvD in other ocular disease types are limited, it is unclear if it is a pathognomonic sign of GON. Lee et al. reported the presence of CMvD (34.1%) in eyes with compressive optic neuropathy (CON) and different features of CMvD in CON versus GON eyes^24^, suggesting that CMvD can develop in any form of optic neuropathy and the characteristics of CMvD should be carefully evaluated to differentiate among various optic neuropathy types.

In our study, eyes with NAION had larger area and wider angular width of CMvD, which was located closer to the temporal region than eyes with NTG (Table 1, Fig. 1). These characteristics can be explained by the pathophysiological differences between NAION and NTG. NAION is presumed to result from a vascular insufficiency or infarct within the retrolaminar portion of the ONH that is supplied by the SPCA^2–4,25^. Acute nonperfusion or hypoperfusion of the SPCA may cause severe and broad ischemic damage in the distal branches, which was noted as having CMvD with larger area and angular width in the eyes with NAION. However, NTG is a chronic progressive optic neuropathy caused by mechanical stress and vascular insufficiency on the ONH. The lamina cribrosa (LC) is the most important site of glaucomatous mechanical and ischemic damage. The superior and inferior parts of the LC contain larger pores and less connective tissue support for the passage of ganglion cell axons than the nasal and temporal parts^26^. A larger laminar pore passes more nerve fiber bundles and has higher metabolic needs. Because a chronic ocular blood flow deficiency in the ONH induces a shortage of adenosine triphosphate, the major energy carrier in axoplasmic flow^27^, ischemic insult can be exaggerated at the superior and inferior regions of the LC, which can result in CMvD locations among NTG eyes as seen in this study.

On the contrary, another hypothesis may explain different clinical features of CMvD observed in our NAION and NTG patients. The ischemic damage to prelaminar ONH tissue and parapapillary choroid supplied by the SPCA may restrict the axonal transport of neurotrophic factors by mitochondria due to hypoxia and by releasing toxic substances that may also have negative effects on axonal function^28^. This, in turn, may promote the apoptosis of remaining RGCs and contribute to progressive axonal loss^28^. Therefore, CMvD detected in the parapapillary choroid may be secondary to the axonal damage and subsequent decline in the metabolic needs at the corresponding ONH locations of axonal loss. The different location and size of CMvD noted in eyes with NAION and NTG may reflect different patterns of primary neuronal damage from ischemia or hypoperfusion, which may lead to secondary loss of microvasculature at various locations of parapapillary choroid in the form of CMvD.

Choroidal VD within the β-PPA was significantly reduced in eyes with NAION and NTG compared to healthy control eyes. However, despite the discrepancy in CMvD characteristics between NAION and NTG eyes, there was no significant difference in the choroidal VD within the β-PPA between them. Our results differed from those of Fard et al., who reported that choroidal VD was significantly reduced in eyes with primary open-angle glaucoma compared to eyes with NAION but that it did not differ significantly between NAION and healthy control eyes^29^. This discrepancy may be explained by differences in study design and methodology for measuring choroidal VD. Unlike the previous study, we included only eyes with β-PPA and measured choroidal VD within the β-PPA, characterized by the absence of retinal pigment epithelium and few superficial retinal vessels; this could minimize the possibility of signal attenuation and the effect of projection artifacts^30^.

In the superficial retinal layer, we also found a discrepancy in global pVD between NAION and NTG eyes. Global pVD was significantly lower in eyes with NAION than in eyes with NTG (Table 1) despite VF MD severity being matched between the two groups. Liu et al. reported that the decrease in pVD was more prominent in eyes with NAION than in eyes with POAG even with similar damage in pRNFLT and VF MD^11^. These findings in the superficial retinal layer are consistent with the extensive ischemic changes in the deep retinal layer represented by the larger area and wider angular width of CMvD in eyes with NAION. The most common systemic disease associated with NAION is hypertension (present in 50% of patients)^31,32^. In a recent study, hypertensive stress is likely to involve vessels in the superficial retinal layer while other conditions such as diabetes involve vessels in the deep retinal layer^33^. Therefore, systemic risk factors, such as hypertension, may have contributed to the reduction of pVD in the superficial retinal layer in eyes with NAION and the differences of pVD between the two diseases. Regionally, temporal pVD was significantly lower in eyes with NAION than in those with NTG (Fig. 2). Since the cilioretinal arteries arise from the SPCA, eyes with NAION may show lower temporal pVD than those with NTG. This may be also related to the temporal distribution of CMvD in eyes with NAION as seen in our study.

Previous studies suggested various discrimination points to distinguish between NAION and GON. The ONH structure (e.g. Bruch’s membrane opening with minimal rim width, rim area, cup-to-disc ratio, and cup volume) differs significantly between NAION and GON eyes^34–37^. Greater LC depth and curvature are characteristic of eyes with GON compared to those with NAION^7,34,38^. Moreover, the RNFL patterns and ganglion cell-inner plexiform layer defects differ between NAION and GON eyes^39,40^. Despite many structural discrimination points, NAION and GON can manifest similar patterns; therefore, the availability of additional discrimination factors would be helpful. In our study, the multivariable logistic regression revealed that CMvD area and location were useful for differentiating NAION from NTG eyes. To our knowledge, no previous study has demonstrated the discriminatory characteristics of the deep retinal layer microvasculature including CMvD in eyes with NAION; therefore, our findings should offer additional perspective for differentiating between NAION and NTG.

There are several limitations to this study. The superficial blood vessels project to deeper tissues and interfere with the accurate visualization of the choroidal microvasculature despite current OCTA providing algorithms for the projection and removal of artifacts. Our results should be interpreted with caution considering this technical limitation of OCTA imaging. Although we reported good agreement and reproducibility of CMvD parameters, determining CMvD can be subjective. All NTG patients were using anti-glaucoma eye drops, which may have affected the ocular blood flow. The possible confounding effect of these medications on the retinal and choroidal microvasculature should be considered when interpreting our findings. Although age was matched between NAION and NTG eyes, older age was one of the statistically significant factors associated with the diagnosis of NAION in our univariable logistic regression analysis. This finding should be interpreted in the context of selection bias during subject matching. Since age was matched among NAION, NTG and healthy eyes after VF MD was first matched between NAION and NTG eyes, age matching among three groups could have introduced selection bias. In addition, relatively wide range of age (± 5 years) for matching among three groups may induce imperfect matching. The applicability of our findings to the eyes with NAION and glaucoma in the general population is limited as; (1) a large proportion of eyes with NAION are excluded due to the absence of β-PPA, (2) most of the NTG cases in the present study have advanced VF damage owing to VF severity being matched with NAION eyes, and (3) relatively small number of patients are included in each diagnostic group. Another limitation is that all of our patients were of Korean ethnicity; thus, it may not be possible to generalize our results to other ethnic groups. Our study did not compare the clinical characteristics of CMvD between primary open-angle glaucoma (POAG) and NTG eyes vs NAION eyes. It is possible that POAG eyes may differ from NTG eyes as well as NAION eyes with respect to the clinical characteristics of CMvD since the pathogenesis of POAG may be more linked to IOP-related mechanical damage than microvascular insufficiency and therefore different from that of NTG or NAION. Future studies with larger sample size are needed to elucidate on this topic. Lastly, due to retrospective nature of this study, there was no information available on the axial length (AL) in eyes with NAION, which can affect the microvasculature measurement of peripapillary retina. Therefore, the impact of AL on the OCTA measurements should be considered when interpreting our study results.

In conclusion, CMvD in eyes with NAION had larger area and wider angular width and was located closer to the temporal region than CMvD in eyes with NTG. The factors associated with the discrimination of NAION from NTG were CMvD area and location after the adjustment for superficial pVD. Different characteristics of CMvD observed using OCTA may provide an alternative approach to differentiating glaucoma from NAION.
